# Deep learning for real-time auxiliary diagnosis of pancreatic cancer in endoscopic ultrasonography

**DOI:** 10.3389/fonc.2022.973652

**Published:** 2022-10-07

**Authors:** Guo Tian, Danxia Xu, Yinghua He, Weilu Chai, Zhuang Deng, Chao Cheng, Xinyan Jin, Guyue Wei, Qiyu Zhao, Tianan Jiang

**Affiliations:** ^1^Department of Ultrasound Medicine, The First Affiliated Hospital, Zhejiang University School of Medicine, Hangzhou, China; ^2^Key Laboratory of Pulsed Power Translational Medicine of Zhejiang Province, Hangzhou, China; ^3^State Key Laboratory for Diagnosis and Treatment of Infectious Diseases, Collaborative Innovation Center for Diagnosis and Treatment of Infectious Diseases, First Affiliated Hospital, Zhejiang University School of Medicine, Hangzhou, China; ^4^Department of Clinical Pharmacy, The First Affiliated Hospital, Zhejiang University School of Medicine, Hangzhou, China; ^5^Zhejiang Provincial Key Laboratory for Drug Evaluation and Clinical Research, Hangzhou, China; ^6^Zhejiang University Cancer Center, Hangzhou, China

**Keywords:** deep learning, endoscopic ultrasonography, diagnosis, ultrasonography, pancreatic lesion

## Abstract

In recent year, many deep learning have been playing an important role in the detection of cancers. This study aimed to real-timely differentiate a pancreatic cancer (PC) or a non-pancreatic cancer (NPC) lesion *via* endoscopic ultrasonography (EUS) image. A total of 1213 EUS images from 157 patients (99 male, 58 female) with pancreatic disease were used for training, validation and test groups. Before model training, regions of interest (ROIs) were manually drawn to mark the PC and NPC lesions using Labelimage software. Yolov5m was used as the algorithm model to automatically distinguish the presence of pancreatic lesion. After training the model based on EUS images using YOLOv5, the parameters achieved convergence within 300 rounds (GIoU Loss: 0.01532, Objectness Loss: 0.01247, precision: 0.713 and recall: 0.825). For the validation group, the mAP0.5 was 0.831, and mAP@.5:.95 was 0.512. In addition, the receiver operating characteristic (ROC) curve analysis showed this model seemed to have a trend of more AUC of 0.85 (0.665 to 0.956) than the area under the curve (AUC) of 0.838 (0.65 to 0.949) generated by physicians using EUS detection without puncture, although pairwise comparison of ROC curves showed that the AUC between the two groups was not significant (z= 0.15, p = 0.8804). This study suggested that the YOLOv5m would generate attractive results and allow for the real-time decision support for distinction of a PC or a NPC lesion.

## Introduction

Accurate diagnosis of pancreatic masses is important for the best treatment strategy (1). Pancreatic cancer, including pancreatic ductal adenocarcinoma (PDAC) and pancreatic acinar cell carcinoma (PACC), is a highly deadly disease with high mortality and poor prognosis. The 5-year overall survival rate was only 5% (2, 3). Surgical resection was considered the curative treatment, but only 15-20% of patients were eligible for surgery due to the late-stage diagnosis (4). The most common EUS imaging features of PC presented a heterogeneous hypoechoic mass of the pancreas, with a predominantly solid mass. The lesion was irregularly bordered (5). Other non-pancreatic cancer (NPC) diseases mainly include pancreatitis, pancreatic neuroendocrine tumors (PNETs), pancreatic pseudocyst (PPC), pancreatic serous cystadenoma (SCA), solid pseudopapillary neoplasm (SPN), and intraductal papillary mucinous neoplasm (IPMN).

In clinical practice of pancreatic lesion examination (6, 7), it could be subjective for the application of relevant items to appraise the lesion. Based on the deep learning, target detection algorithm may objectively assess whether it was a PC or a NPC lesion through the endoscopic ultrasonography (EUS) image. However, manual image labeling for target detection would be time-consuming when labeling a large number of images. Moreover, labeling would be not real-time and it was difficult to provide immediate diagnosis information during the lesion detection. There were some factors disturbing the examination process, such as the breathing or patient’s position changes. Detection and classification of the pancreatic lesion is challenging owing to the heterogeneity of the pancreas. Additionally, there were few studies real-timely examining pancreatic lesion during EUS image. Recently, YOLO (You Only Look Once) series in target detection contained YOLOv1, YOLOv2, YOLOv3, YOLOv4 and YOLOv5 (8). They could directly extract features from the images and predict the categories and positions of objects through regression analysis. Among them, YOLOv5 has high real-time performance and accuracy. Based on urgent clinical need, we used a fully automatic YOLOv5 model for the EUS image of pancreatic lesions. It could provide effective and real-time monitoring, which may automatically locate the lesion, and distinguish that it was a PC or a NPC lesion.

## Materials and methods

### Data collection and preparation

**Figure 1:**
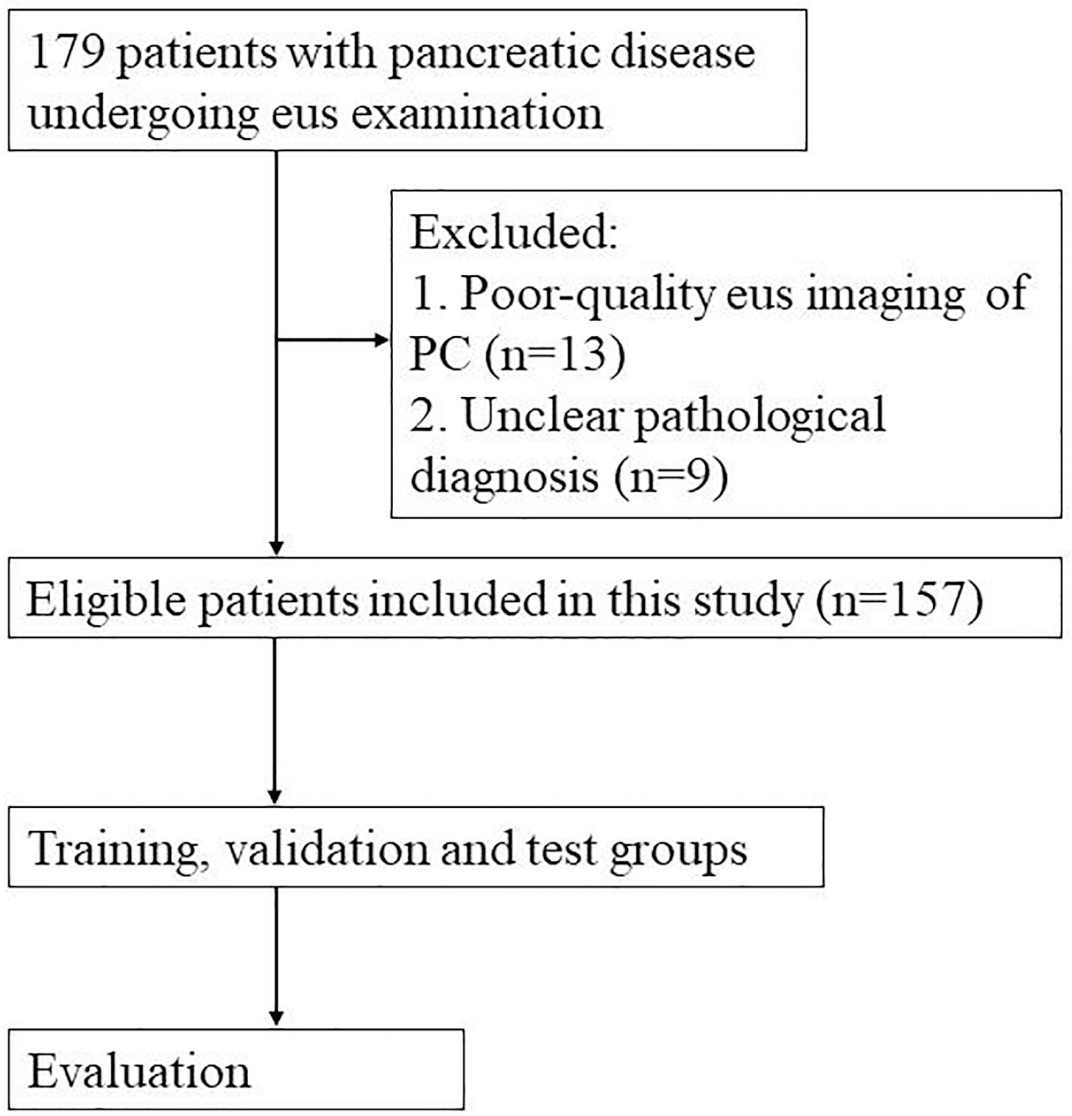
Flowchart depicting the selection process for this study.

This study was conducted for pancreatic lesions examinations using an orally curved linear array echoendoscope (Olympus Ltd, Tokyo, Japan). The pancreatic lesions were sampled with a 22-gauge aspiration needle (Wilson Cook Medical, Bloomington, United States). The flowchart depicting the selection process for this study was shown in **Figure 1**. Patients in this study were eligible for the following characteristics. Firstly, patient underwent EUS examinations of the pancreas. Secondly, the reference for pancreatic lesion (such as PC and PNETs) in the study was determined by surgical or biopsy pathology (9, 10). Some pancreatic cyst and pancreatitis were diagnosed based on a combination of clinical symptoms, laboratory tests and imaging examination. For example, many patients with cystic lesions of the pancreas were asymptomatic and a few of them may have symptoms such as pain, bleeding, jaundice or a palpable mass. Serum CA19-9 level may be increased in malignant cystic lesions, and amylase and lipase levels may be elevated in the presence of pancreatitis. In addition, imaging examination (including CT, MRI, MRCP, EUS) and cyst fluid analysis were important in the classification of cystic lesions of the pancreas (11). The diagnostic criteria for acute pancreatitis mainly included 1) a history of persistent upper abdominal pain, 2) the serum amylase or lipase level 3 times higher than the upper limit of normal range, and 3) imaging findings of acute pancreatitis (12). The diagnostic criteria for chronic pancreatitis mainly contained 1) typical and atypical imaging features, 2) histopathological changes, 3) symptoms of recurrent upper abdominal pain, 4) abnormal serum or urinary pancreatic enzyme levels, 5) abnormal pancreatic exocrine function, and 6) a history of chronic alcohol abuse (13). Exclusion criteria were as follows: a. absence of EUS imaging of the pancreas or poor-quality EUS imaging of the pancreas for other reasons, and b. unclear pathological diagnosis.

A total of 179 patients with pancreatic disease undergoing EUS examination at the First Affiliated Hospital of Zhejiang University from January 2017 to May 2022 were included in this study. Thirteen patients with poor-quality EUS imaging of PC and 9 patients with unclear pathological diagnosis were excluded. The final 157 patients were included in this study. A total of 1213 images were from these cases, and about 1-20 pictures were extracted from the EUS video of each patient. Of these, 102 patients had pathologically confirmed pancreatic cancer, including 101 PDAC and 1 PACC. Fifty-five patients had non-pancreatic cancer, including 34 pancreatitis, 13 PNET, 5 PPC, 2 SCA and 1 IPMN. The enrolled cases were randomly grouped into training, validation and test groups, with a ratio of approximately 4:1:1, respectively. This retrospective study was approved by the ethics committee of the First Affiliated Hospital of Zhejiang University, and informed consent was obtained. All included images were extracted and checked by two investigators with over 10 years of EUS experience. The two investigators determine whether a PC (marked by the number 1) or a NPC (marked by the number 2) lesion was present based on the characteristics of the EUS image. For example, EUS imaging in chronic pancreatitis often showed an atrophied or localized enlargement of the pancreas. The pancreatic parenchyma was echogenically thickened and enhanced. The margins of the pancreas and the walls of the pancreatic duct were irregular. EUS imaging in acute pancreatitis indicated one or more dark areas of fluid with well-defined borders within the pancreatic tissue. The interior was anechoic or multiroom-like with posterior echogenicity. Most PNETs presented well-defined, regular hypoechoic masses during EUS imaging (14, 15). Before network training, Labelimage software was used for target detection *via* manual image labeling. We used rectangular boxes to manually draw regions of interest (ROIs), and mark the PC and NPC lesions. The quality of the ROI contours was crosschecked by two investigators. The manually annotated.xml file needed to be converted into.txt file, and then saved into train.txt and validation.txt file.

### Deep learning algorithm

YOLO algorithms could obtain features of the EUS images, and classify and locate the objects through regression analysis. YOLOv5 algorithms include input, backbone, neck, and prediction layers (16). The input part is mainly used for data enhancement, adaptive image scaling, and anchor frame calculation. The backbone part is the backbone network, which could extract the key information from the input samples using the cross-stage-partial-connections (CSP) structure. The neck part uses the information obtained from the backbone part to strengthen multi-scale feature fusion through feature pyramid network (FPN) and path aggregation network (PAN) structures. The prediction part is used for forecasting and obtaining values such as GIOU_Loss. According to the depth of the network and the width of the feature map, YOLOv5 is divided into four different structures of YOLOv5s, YOLOv5m, YOLOv5l, and YOLOv5x. In this study, YOLOv5m was used as the algorithm model.

The learning rate was set to 0.0005, the training epochs to 300 and the batch size to 2. After YOLOv5m model training and test running, this study used the following parameters to evaluate the results of target detection, such as Generalized Intersection of Union (GIoU) Loss, Objectness Loss, precision, recall, multi-category average precision under 0.5 intersection ratio threshold (mAP@0.5), multi-category average precision under changing intersection ratio threshold (mAP@0.5:0.95). Among them, recall means the probability that the PC cases in all patients are correctly predicted. Precision means the probability that the PC cases in all patients diagnosed by YOLOv5m are correctly predicted. For the output, if a lesion existed in the image/video, the model will automatically frame the location of the lesion with a rectangle and prompt what is the probability that it is a PC lesion. In the output image, the number 1 meant a PC lesion, while the number 2 represented a NPC lesion.

### Statistical analysis

Python 3.6 was used in this study *via* the Windows 10 system based on Intel Core i7-10870H CPU, and YOLOv5m was executed under the PyTorch framework. The model was running based on NVIDIA GeForce RTX 2060 with 32GB memory. Receiver Operating Characteristic (ROC) analysis was performed using the DeLong test of MedCalc 20.100 software to compare the accuracy of the physician and this model in discriminating between PC and NPC lesions in pancreatic EUS images in test group. For all estimations in this study, P < 0.05 was deemed statistically significant.

## Results

### Summary of the included participants

For the 102 PC (101 pancreatic ductal adenocarcinoma, 1 pancreatic acinar cell carcinoma) and 55 NPC patients (34 pancreatitis, 13 PNET, 5 PPC, 2 SCA, and 1 IPMN), the summary of the enrolled patients was shown in **Table 1**. The mean age was 63.36 ± 0.87 years for PC group and 57.47 ± 1.5 years for NPC group. For the PC group, the lesions in 51 cases were located at the head, neck and uncinate of pancreas, 19 cases at the body of pancreas, and 32 cases at the tail of pancreas. The mean size of the lesions in the PC group was 2.72 ± 0.92 cm. And for the NPC group, the lesions in 32 cases were located at the head, neck and uncinate of pancreas, 7 cases at the body of pancreas, and 16 cases at the tail of pancreas. The mean size of the lesions in the NPC group was 2.53 ± 0.31 cm.

**Table 1:** The summary of the enrolled patients.

	Pancreatic cancer	Non-pancreatic cancer
Total number of patients	102	55
	101 (PDAC)	34 pancreatitis
	1 (PACC)	13 (PNET)
		5 (PPC)
		2 (SCA)
		1 (IPMN)
Sex, n		
Male	64	35
Female	38	20
Age (y)		
Mean ± SD	63.36 ± 0.87	57.47 ± 1.5
Localization		
Head (neck and uncinate)	51	32
Body	19	7
Tail	32	16
Lesion size (cm)		
Mean ± SD	2.72 ± 0.92	2.53 ± 0.31

PDAC, pancreatic ductal adenocarcinoma;

PACC, pancreatic acinar cell carcinoma;

PNET, pancreatic neuroendocrine tumor;

PPC, pancreatic pseudocyst;

SCA: pancreatic serous cystadenoma.

IPMN, intraductal papillary mucinous neoplasm.

### Network model training phase

**Figure 2:**
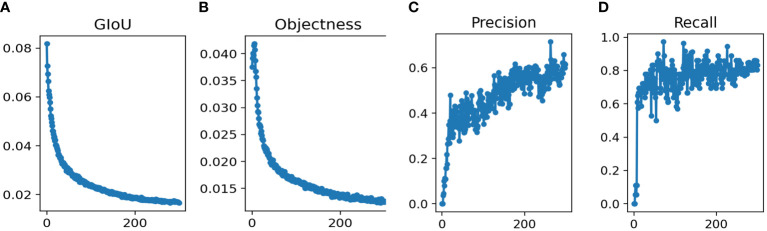
Performance of Yolov5s network structures in the training datasets for pancreatic tumor diagnoses **(A)** GIoU Loss **(B)** Objectness Loss **(C)** Precision **(D)** Recall.

The total runtime was 18h 11min. We used the YOLOv5m model for the training epochs 300 in this study. After training the model, the training results were shown in **Figure 2**. The parameters achieved convergence within 300 rounds (GIoU Loss: 0.01532, Objectness Loss: 0.01247, precision: 0.713 and recall: 0.825).

### Network model validation results

**Figure 3:**
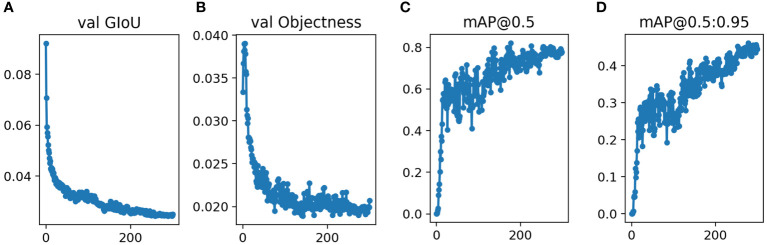
Performance of Yolov5s network structures in the validation datasets for pancreatic tumor diagnoses **(A)** GIoU Loss **(B)** Objectness Loss **(C)** mAP0.5 **(D)** mAP@0.5:0.95.

After training the model, the validation data was input into the model. For the validation group, **Figure 3** showed that the validation results were convergent after 300 epochs in this study. The mAP0.5 was 0.831, and mAP@.5:.95 was 0.512.

### Network model test results and analysis

**Figure 4:**
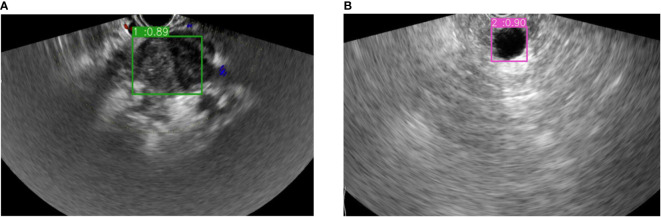
The example of bounding boxes for real-time auxiliary diagnosis in the test datasets. **(A)** Pancreatic cancer prediction and its possibility. **(B)** Non-pancreatic cancer lesion prediction and its possibility.

In addition, this study used this model to differentiate the PC or NPC lesions of the pancreas in test group, and compared the results with those diagnosed by physicians using EUS detection without puncture. The ROC curve analysis seemed to reveal a more AUC of 0.85 (0.665 to 0.956) with the sensitivity 95% and specificity 75% in this model while AUC of 0.838 (0.65 to 0.949) with the sensitivity 80% and specificity 87.5% for physicians. However, pairwise comparison of ROC curves showed that the AUC between the two groups was not significant (z= 0.15, p = 0.8804). The examples of the prediction for PC or NPC lesions in EUS images were shown in **Figure 4**, which were consistent with the pathological results.

## Discussion

In this study, we showed that the diagnostic performance of YOLOv5m architecture was relatively high and robust. It could quickly and accurately process data sets in the test groups. The target recognition algorithm included target recognition and target positioning. During target detection, it usually searched the boxes of different sizes from all the pixels repeatedly, which was inefficient. Through the two-stage target detection algorithm, RCNN first found several selected regions from the primary image, and then recognized the targets for the selected regions, in which the efficiency of training and testing was low. The Yolo series was based on multiple feature maps, which directly output the location and category of the detected target.

In recent years, many deep learning applications have become important ways to aid in the detection of cancer, including liver (17, 18), eye (19), skin (20), breast (21), colorectum (22), kidney (23), and spine (24) through computed tomography (CT), and magnetic resonance imaging (MRI), and ultrasonography. CT played an important role in the diagnosis of PC. Chu et al. reported that in the retrospective study of 255 training cases and 125 validation cases based on the preoperative CT radiomics features, the overall accuracy was 99.2% (124/125), and AUC was 99.9% (25). Luo et al. used a CNN-based DL to estimate the pathological grading of PC *via* CECT images. It predicted the grading more accurately than the radiologists (73.12% vs. 58.1%) (26). But there was a risk of radiation exposure in these patients, and the sample size was small.

For the deep learning method, Naito et al. used CNN model based on the pancreatic EUS-FNB specimens and blood test to show high area under the receiver operating characteristic curve (AUROC) of 0.984, accuracy of 0.9417, sensitivity of 0.9302 and specificity of 0.9706 (27). Regarding conventional EUS, previous studies suggested that the sensitivity for detection of PC was 0.9 (range: 0.7-1) (28). In a multicenter study of EUS elastography for differential diagnosis of focal pancreatic masses, it indicated a sensitivity of 0.934, a specificity of 0.66, a positive predictive value of 0.925, a negative predictive value of 0.689, an overall accuracy of 0.854, and an AUROC of 0.854, respectively (29). But during the procedure of EUS-guided fine needle aspiration (FNA), it could cause the adverse reactions such as perforation, bleeding, pancreatic fistula, pancreatitis, or tumor dissemination. If EUS worked with DL, they could effectively reduce these complications. Furthermore, DL may assist physicians to reduce the rate of misjudgment or missed diagnosis.

This study focused on the real-time distinction of a PC or a NPC lesion during the EUS guidance. It showed attractive performance on pancreatic lesion diagnosis, which could become a promising assistant in the clinical practice. It was meaningful for patients with negative EUS-FNA results, which assist physicians to reminder a repeat EUS-FNA, follow-up, or referral to surgery. However, this study has some limitations. Firstly, more training will be imperative to differentiate a PC or a NPC lesion to improve the clinical effectiveness of this model. The sample sizes for the validation and test groups were small, and all patients were obtained from a single institution. This may account for the negative AUC results produced by the model when compared to those diagnosed by physicians using the EUS examination without puncture. Further studies with large-scale patients including other institutions with each disease will be needed to improve the robustness of this model. Secondly, this study only trained YOLOv5m, while YOLOv5l and YOLOv5x were not trained due to hardware limitations.

In short, the YOLOv5m would generate promising results and could have the advantage of real-time detection. These findings would be helpful to reduce misdiagnosis between PC and NPC lesions, and improve patient prognosis. In the future, we will further develop the model on more data obtained from other institutions to estimate its performance.

## Data availability statement

The original contributions presented in the study are included in the article/supplementary material. Further inquiries can be directed to the corresponding author.

## Ethics statement

This retrospective study was approved by the ethics committee of the First Affiliated Hospital of Zhejiang University, and informed consent was obtained. The patients/participants provided their written informed consent to participate in this study. Written informed consent was obtained from the individual(s) for the publication of any potentially identifiable images or data included in this article.

## Author contributions

TJ contributed to the conception and design of the article. GT analyzed and wrote the manuscript. DX, YH, WC, and ZD revised the manuscript. All authors contributed to manuscript data collection, and approved the submitted version.

## Funding

This study was supported by the Development Project of National Major Scientific Research Instrument (82027803); National Natural Science Foundation of China (81971623); Key Project of Natural Science Foundation of Zhejiang Province (LZ20H180001); Zhejiang Provincial Association Project for Mathematical Medicine (LSY19H180015).

## Conflict of interest

The authors declare that the research was conducted in the absence of any commercial or financial relationships that could be construed as a potential conflict of interest.

## Publisher’s note

All claims expressed in this article are solely those of the authors and do not necessarily represent those of their affiliated organizations, or those of the publisher, the editors and the reviewers. Any product that may be evaluated in this article, or claim that may be made by its manufacturer, is not guaranteed or endorsed by the publisher.
